# Attenuated Disease in SIV-Infected Macaques Treated with a Monoclonal Antibody against FasL

**DOI:** 10.1155/2007/93462

**Published:** 2007-12-09

**Authors:** Maria S. Salvato, C. Cameron Yin, Hideo Yagita, Toshihiro Maeda, Ko Okumura, Ilia Tikhonov, C. David Pauza

**Affiliations:** ^1^Institute of Human Virology, University of Maryland Biotechnology Institute, Baltimore, MD 21201, USA; ^2^Department of Pathology and Laboratory Medicine, School of Medicine and Public Health, University of Wisconsin, Madison, WI 53705, USA; ^3^Department of Hematopathology, MD Anderson Cancer Center, University of Texas, Houston, TX 77030, USA; ^4^Department of Immunology, Juntendo University School of Medicine, Tokyo 113-8421, Japan; ^5^The Chemo-Sero Therapeutic Research Institute (Kaketsuken), Kumamoto 860-8568, Japan

## Abstract

Acute SIVmac infection in macaques is accompanied by high levels of plasma viremia that decline with the appearance of viral immunity and is a model for acute HIV disease in man. Despite specific immune responses, the virus establishes a chronic, persistent infection. The destruction of CD4+ and CD4- lymphocyte subsets in macaques contributes to viral persistence and suggests the importance of mechanisms for depleting both infected and uninfected (bystander) cells. Bystander cell killing can occur when FasL binds the Fas receptor on activated lymphocytes, which include T and B cell subpopulations that are responding to the infection. Destruction of specific immune cells could be an important mechanism for blunting viral immunity and establishing persistent infection with chronic disease. We inhibited the Fas pathway in vivo with a monoclonal antibody against FasL (RNOK203). Here we show that treatment with anti-FasL reduced cell death in circulating T and B cells, increased CTL and antibody responses to viral proteins, and lowered the setpoint viremia. By blocking FasL during only the first few weeks after infection, we attenuated SIVmac disease and increased the life span for infected and treated macaques.

## 1. INTRODUCTION

In 1991, Amiesen and Capron proposed that inappropriate induction of activation-induced cell death (AICD) was a major mechanism for depleting CD4+ T cells during HIV disease [1] and they demonstrated apoptosis in PBMC from HIV-infected individuals [2]. A molecular mechanism for AICD was demonstrated in 1995, involving FasL (known then as APO-1) binding to its receptor [3], and FasL-mediated apoptosis was elevated in PBMC from HIV-infected individuals [4].

Activation-induced cell death (AICD) is a feature of normal physiology and can be demonstrated in vitro. T cells that are stimulated by ligating their T cell receptor (TCR) and then restimulated a few days later, will die by apoptosis [5]. When AICD affects mature, circulating T cells, it is termed peripheral deletion and this mechanism can extinguish the response to a particular antigen by deleting all lymphocyte clones with that receptor specificity. Peripheral deletion often occurs after exposure to superantigens, where we observe the loss of specific V-beta T cell subpopulations.

Many viruses and bacteria exploit lymphocyte depletion mechanisms in order to establish persistent infections. By eliminating pathogen-specific immunity, microbes can avoid detection and elimination. A classic example is lymphocytic choriomeningitis virus infection in mice. Some strains of LCMV are acutely lethal, and the CTL response is a major part of the immune pathology. Nonlethal, persisting strains of LCMV trigger the deletion of virus-specific CTL, thus reducing pathology and allowing for chronic infection [6, 7]. We believe that HIV is similar, in that infection promotes a mechanism for deleting antiviral immune cells. In HIV disease, immune depletion is not limited to antiviral responses and eventually spreads to disrupt immunity against a number of intercurrent pathogens. The result increased susceptibility to opportunistic infections that become major factors in disease and death.

Viral proteins have been implicated in the regulation of T cell activation and Fas-mediated killing. Both the HIV-1 Tat and Env proteins can activate cells and induce Fas-mediated killing [8, 9]. Tat protein activates the Fas ligand promoter [10], and soluble Tat causes production of FasL and another death ligand (TRAIL) in monocyte/macrophages or dendritic cells [11–13]. Chemically-inactivated virions trigger both T cell activation and apoptosis [14]. Env glycoprotein alone upregulates FasL [15], although it is controversial whether this occurs with monomeric gp120 or needs CD4 crosslinking. Direct binding to CCR5 also induced FasL [16]. HIV disease is characterized by extensive lymphocyte activation with elevated expression of Fas receptor (CD95) on a majority of circulating T cells. These activated cells are killed when FasL binds [4, 17]. FasL itself is upregulated during HIV infection [18, 19] and is especially high on antigen-presenting cells [20, 21] where it is poised to kill CD4+ T cells during their initial encounter with antigen.

Apoptosis was evident in lymph nodes from macaques acutely infected with SIV and the proportion of apoptotic cells was highest for rapid progressors [22] and we showed that macaques with pre-existing, high levels of FasL-mediated cytotoxicity for human B lymphoblastoid cell line (B-LCL) targets, became rapid progressors after SIVmac infection [23]. In the present study, we tested the hypothesis that FasL-mediated cell death is important for SIV disease in macaques, by injecting a monoclonal antibody that neutralizes FasL [24] during the interval of acute infection.

## 2. RESULTS

**Figure 1:**
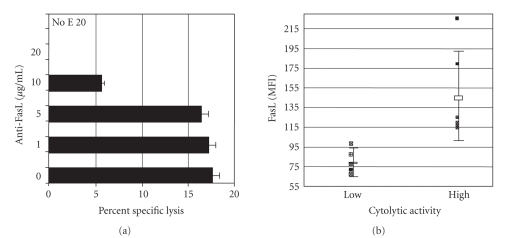
The anti-FasL monoclonal antibody blocks MHC-unrestricted cytolysis and stains FasL on rhesus PBMC. (Panel A) Anti-FasL, the humanized NOK2 monoclonal (RNOK203), blocked MHC-unrestricted cytolysis in vitro using rhesus or human PBMC as effectors and human or rhesus B-LCL targets that expressed SIV envelope glycoprotein (transfected with pCDNA3/SIVenv). The four-hour chromium-release assay was done in the presence of 1 mM EGTA and varying amounts of anti-FasL at an E:T ratio of 50:1 as described [23]. Specific lysis was blocked completely at 20 μg/mL of monoclonal antibody (shown) and not with a similar concentration of isotype control antibody (not shown). When no effector cells were added (No E), there was no specific lysis showing that 20 μg/mL anti-FasL does not induce cell death on its own. These experiments were done 3 times with rhesus PBMC, 3 times with human PBMC, and 2-3 times cross species with the same results. The error bars represent the variation in 3 different experiments using rhesus cells. (Panel B) MHC-unrestricted cytolytic activity correlated with the mean fluorescence intensity (MFI) of cell surface FasL on circulating PBMC. PBMC specimensfrom rhesus macaques were screened by the MHC-unrestricted cytolysis assay as described [23]. Results from 10 rhesus macaques are shown here. Macaques were divided into high (≥15% specific lysis) and low (<10% specific lysis) groups, and stained with the anti-FasL antibody in an indirect immunofluorescence assay. The individual points, mean values, and standard deviations are shown for animals classified as having low or high cytolytic activity. Macaques with high levels of MHC-unrestricted cytolytic activity and correspondingly high levels of cell surface FasL were selected for this study.

The FasL-specific, recombinant monoclonal antibody RNOK203 [24] inhibited MHC-unrestricted cytotoxicity in vitro (Figure 1(a)) and MHC-unrestricted cytotoxicity was correlated with the levels of cell surface FasL on PBMC from virus-naïve macaques (Figure 1(b)). Pilot studies with anti-FasL at 4 mg/kg showed no noticeable impact on T or B cell counts in healthy monkeys (not shown). Injection of RNOK203 into a control (uninfected) macaque caused a transient decrease in MHC unrestricted cytolysis of human B-LCL targets (Table 1), with cytotoxicity returning to normal a few weeks after antibody treatment, compared to stable levels of B-LCL cytolysis in one macaque treated with a control IgG and one untreated animal.

We screened rhesus macaques and selected eight animals (from a total of 19) with both high levels of FasL-mediated, MHC-unrestricted cytotoxicity, and elevated FasL expression on PBMC. Based on the potency of RNOK203 for blocking FasL-mediated cytotoxicity in vitro, we determined that 4 mg/kg body weight was an appropriate single dose for juvenile macaques. Selected animals received either anti-FasL or human IgG1 (isotype control). All animals received five intravenous injections of antibody in saline at 1 week before, the time of, then 1, 2, and 3 weeks after SIV inoculation. Each animal was inoculated by intravenous injection, with 40 minimal animal infectious doses of SIVmac239 [25]; the time of virus inoculation was defined as week 0. We confirmed the impact of anti-FasL in vivo, by a reduction in the frequency of dying lymphocytes in blood. Assays for both MHC Class I-restricted CTL activity against the SIV p27 Gag and virus-binding serum antibodies characterized the impact of RNOK203 on viral immunity. We measured acute and set-point plasma viremia to characterize the course of infection; survival time was also measured.

**Figure 2:**
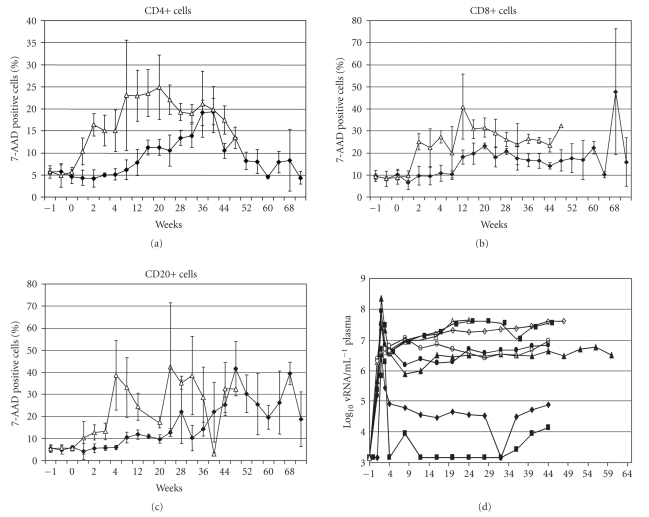
Inhibition of cell death and reduced plasma vRNA after treatment with anti-FasL. Macaque PBMC specimens were collected 1 week before (−1) and at regular intervals after SIVmac239 infection (that occurred at week 0). The animals were treated with RNOK203 or a control IgG1 (Sigma #I-3889). Panels (a)–(c) show the proportion of dying (positive for 7-AAD staining) lymphocytes in the CD4+, CD8+, or CD20+ subsets. Open symbols represent the average values among four macaques treated with control IgG. Closed symbols represent the average values among four macaques treated with anti-FasL. Error bars show the standard error of the mean for each time point. The absolute cell counts for panels (a)–(c) are summarized in Table 2. Panel D shows the plasma SIVmac vRNA levels for each animal determined by b-DNA assays (conducted by Bayer Diagnostics, Emeryville, CA). The two animals with lowest set-point RNA were also the two that survived longest (Figure 4). Open symbols represent control animals and closed symbols represent macaques treated with anti-FasL. The limit of detection for this assay was 10^3^vRNA copies per mL of plasma.

The frequency of circulating lymphocytes that incorporate 7-amino actinomycin D was reduced by treatment with anti-FasL for CD4-positive (Figure 2(a)) and CD8-positive T cells (Figure 2(b)), and for B cells (Figure 2(c)). Control macaques showed a sharp increase in the frequency of dying T and B cells within 1 week after infection; these values increased to peak levels of around 25% for CD4+ T cells, 30% for CD8+ T cell, and 40% for CD20+ B cells. Anti-FasL-treated animals had only baseline levels of dying cells until around 6–8 weeks after SIV infection. Similar results were obtained with monoclonal antibodies against Annexin V, a marker for lymphocyte apoptosis (not shown). By 8 weeks after SIVmac infection (Table 2), the IgG-treated (control) animals had a 49% + 8% of starting CD4 cell counts, compared to an 87% + 36% of starting CD4 T cells in the anti-FasL-treated group (*P* =.03 by t test). Similar effects were noted for CD20+ B cells where control animals had 43% ± 16% compared to the anti-FasL-treated group which had 116% ± 74% of starting B cell counts (*P* =.03). At 8 weeks, there were no significant differences in the CD8+ T cell counts among control and anti-FasL-treated groups.

Lower cell death levels persisted until around 40 weeks after infection. Acute viremia was similar among treated and control groups, but RNOK203 lowered the set-point vRNA levels (from the limit of detection at 10^3^ to 6 × 10^6^ copies per ml), compared to the range of 3 × 10^6^ to 4 × 10^7^ copies per mL for controls. Thus, the reduced lymphocyte cell death seen before 40 weeks was associated with lower vRNA, although some treated animals had viremia in the range of controls.

**Figure 3:**
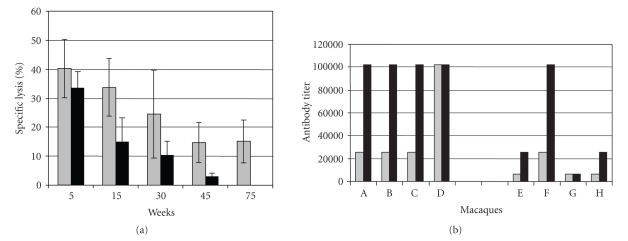
Cellular and humoral immune responses to SIV are enhanced by anti-FasL treatment. MHC-restricted CTL to SIV p27 Gag protein (Panel A) were measured in a standard 4 hours. ^51^Cr-release assay [26]. Targets were syngeneic B-LCL (different lines for each animal) that were either uninfected or infected with VVgag as described [26]. Percent specific lysis at the 50:1 effector to target ratio was plotted. The mean and standard errors of the mean (SEM) are shown for four animals treated with anti-FasL (shaded bars) or four control macaques (solid bars) at the times indicated. The values for CTL activity were consistently higher among macaques treated with anti-FasL compared with controls, but the differences were not significant at 30 weeks after infection. Virus binding titers (Panel B) were measured by ELISA using plates coated with p27 Gag antigen as described [27]. Sera from weeks 12 (shaded bars) or 24 (solid bars) were diluted with normal saline, up to a maximum dilution of 1:100,000. Macaques (A, B, C, D) on the left side of the figure had been treated with anti-FasL, and macaques (E, F, G, H) on the right side had been treated with control IgG. Anti-FasL treatment caused a substantial elevation in virus-binding antibodies in SIV-infected macaques, which have been shown to correlate with reduced disease progression [28].

CTL to p27 Gag protein and virus-binding serum antibodies were measured in treated and control macaques (Figure 3). With effector to target cell ratios of 50, we observed specific lysis of around 40% for anti-FasL-treated and 33% for control macaques by 5 weeks after SIV infection (Figure 3(a)). These modest differences were statistically significant at weeks 15 and 45 (*P* < .05); death of control animals precluded statistical comparisons after 45 weeks. By 24 weeks after SIV infection, all treated animals had virus-binding antibody titers ≥100,000, compared to 1 of 4 animals in the control group (Figure 3(b)). Overall, there was a modest effect of anti-FasL on virus-specific CTL activity, but a pronounced effect on virus-binding antibodies.

**Figure 4:**
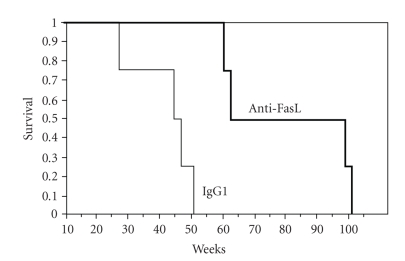
Anti-FasL treatment prolongs survival in SIVmac-infected macaques. The fraction of surviving animals is plotted for up to 102 weeks after SIVmac239 infection. The thin line shows the fraction of animals surviving after control IgG treatment and SIV infection. All control animals died by 52 weeks after SIV infection. The thick line shows the effect of anti-FasL treatment (anti-FasL) in extending survival times. None of the anti-FasL-treated animals had died before 60 weeks after SIV infection, and the last surviving animal lived until 102 weeks. The two longest-lived animals also had lowest viral loads (Figure 2(d)).

Increased viral immunity and lower set-point vRNA levels argue that disease was attenuated after the brief treatment with RNOK203. The fraction of surviving animals (Figure 4) showed a significant difference between anti-FasL-treated animals and control groups. The control group behaved as expected for intravenous SIVmac239 in rhesus macaques [28]; half were dead by 42 weeks and all succumbed by 52 weeks. None of the anti-FasL-treated animals were dead before 60 weeks and the last survivors remained until 102 weeks. The two animals surviving the longest had the lowest set-point viremia, implying that the impact of RNOK203 on virus replication was linked with disease progression and survival.

## 3. DISCUSSION

Some aspects of AIDS pathogenesis remain unsettled. Two recent papers highlighted the conflict between models focusing solely on viral replication as the cause for memory T cell depletion and disease [29] and models that incorporate both virus replication and bystander cell killing [30]. It is important to remember that virally infected cells are resistant to death signals delivered by FasL or TNF-related apoptosis-inducing ligand (TRAIL) [13, 31–34]; these soluble death ligands are induced during virus infection and would promote the depletion of uninfected cells to effectively increase the proportion of infected cells. Thus, observed increases in the relative abundance of infected cells may not be explained solely by virus dissemination.

Bystander cell killing via FasL is likely triggered by high viremia during acute infection, but numerous examples argue that virus replication alone is insufficient for disease. Several macaque species maintain high levels of SIV replication without evident disease [35, 36]. There are indications in these models that reduced lymphocyte apoptosis in vivo and resistance to activation-induced cell death in vitro can lessen bystander cell killing and prevent disease [37, 38]. SIV-infected cells upregulate FasL and can thereby mediate the destruction of virus-specific cytotoxic T cells [34]. At the same time, SIV-infected cells avoid apoptosis by upregulating Bcl-2 [33]. These events explain how high FasL-mediated bystander killing could also lead to high virus loads. The loss of uninfected cells not only depletes T and B cells responding to viral antigens, but also impacts gamma/delta [39] and NK T cell [40] populations that comprise critical innate responses to viral infection and disease. The latter subsets are not usually infected by HIV-1 and are likely depleted by an indirect mechanism, consistent with FasL-mediated death of activated cells.

Mechanisms by which FasL kills activated T and B cells have been described [41, 42], and it is known that this killing can be blocked by FasL antagonists [43, 44]. Activation increases the expression of Fas receptor on T and B cells [3] and even resting cells can be killed by high levels of FasL [45]. We have considered the simplest role for anti-FasL as an inhibitor of T and B cell death, and we documented increases in T and B cell subsets that are consistent with that role. Our model is that anti-FasL is blocking FasL on cell surfaces or on released vesicles and preventing the destruction of effector T and B cells. The surviving effectors eventually destroy virus-infected cells and limit virus spread and disease. However, more complex indirect mechanisms could account for our results. For example, FasL treatment of B cells causes both cell death and down-modulation of IgM production by different pathways [46]. Also, cytotoxic CD4 cells can destroy B cells in a Fas/FasL-dependent manner, but only if the B cells were CD40L-activated and not if they were activated through the antigen receptor [47]. Thus, anti-FasL could also be enhancing effector functions that are indirectly responsible for decreases in viral load or increases in B and T cells.

Antiretroviral therapy during acute infection can modulate the long-term consequences of SIV infection [48, 49], including a reduction in lymphocyte apoptosis [50]. In this intervention study, peak acute viremia was not affected by anti-FasL treatment arguing that virus replication was not the critical factor for disease. An early and transient inhibition of FasL allowed for increased viral immunity, lower viremia in some of the treated animals, and longer survival after SIV infection. In human beings, antiretroviral therapy during acute HIV infection [51] was associated with increased viral immunity but the effect was not durable [52]. With expanding efforts to identify persons with acute HIV infection through population screening [53] and the need to treat children acutely infected through vertical transmission, important opportunities may emerge for combining antiretroviral drugs with modulators of cell killing to reduce disease and prolong the interval for AIDS-free survival.

## 4. METHODS

### 4.1. Animal infections and sampling intervals

Eight rhesus macaques housed at the Wisconsin Regional Primate Research Center, 3-4 years of age, retrovirus free, and foamy-virus free, were selected because they had high MHC-unrestricted lytic activity [23]. Baseline values for lymphocyte subsets, activation markers, and FasL were determined over 3 months, then animals were infected with 40 TCID50 of SIVmac239 as described [28]. Animals were confirmed positive for infection by two independent virus isolation assays and tests for SIV viremia. Venous blood samples were collected weekly from 4 weeks before infection to 5 weeks after infection, then at weeks 7, 10, 12, and 15 after infection, and then approximately every four weeks until euthanasia. The percentage of dying cells in CD4+, CD8+,and CD20+ lymphocyte subsets, plasma vRNA levels, MHC Class I-restricted cytotoxicity, and virus binding antibody titers were determined among other standard characterizations and daily observations. Macaques were euthanized when, in the opinion of the attending veterinarian, they had entered a terminal disease state and euthanasia was required to alleviate unnecessary suffering. The research protocol was approved by the Institutional Animal Care and Use Committee of the University of Wisconsin Graduate School.

### 4.2. Virus stocks and cell lines

The stock of SIVmac239 was stored at –130°C at 400 TCID50/ml as the third passage of a stock originally obtained from Dr. Ron Desrosiers [54]. Recombinant vaccinia viruses (gift from Therion Biologics, Cambridge, MA) were derived from the NYCBH vaccinia strain and contained either no insert or genes from SIVmac251.

Human B-lymphoblast cell lines (B-LCL) were produced by transformation of human PBMC with human Epstein Barr virus (EBV) B95-8 cell supernatant kindly provided by Dr. W. Sugden. B-LCL were maintained in RPMI-1640 (Gibco, Gaithersburg, MD) supplemented with L-glutamine (2 nM), penicillin (50 U/ml), streptomycin (50 U/ml), and 10% fetal calf serum (Harlan Sprague Dawley, Madison, WI), then diluted into wells of 24-well plates and cryopreserved as B-LCL. Rhesus macaque B-LCL were obtained by transforming rhesus PBMC with *Herpes papio* from S594 cell supernatants kindly supplied by N. Letvin, and were stored as viable, frozen cells.

### 4.3. Antibodies and animal treatments

The monoclonal antibody to surface FasL was RNOK203 [55]. The isotype control antibody was human IgG1 (Sigma #I-3889) purified by binding to Protein G agarose beads (Sigma) and eluting in 100 mM glycine HCL (pH 2.7). The concentrated antibody solution was dialyzed against PBS, then stored in buffer at 5 mg/ml. Antibody infusions were 4 mg/kg or for a 3 kg animal, about 12 mg in 2.4 mL for each inoculation. Macaques A, B, C, and D were given anti-FasL and macaques E, F, G, and H were given the control IgG.

Levels of endotoxin in anti-FasL and control IgG antibodies were less than 13 EndotoxinUnits/mL (approximately 2.5 ng/mL determined by an E-toxate kit (Sigma)) and were similar to levels in normal monkey plasma. To insure the safety of antibody infusions, each procedure was preceded by a skin test: 0.2 mL of a 1/1000 dilution of the antibody was inoculated subcutaneously and observed for 10 minutes. This was negative for all animals. As an additional precaution, all antibody infusions were accompanied by injections with antihistamine (Benadril) at 5 mg/kg. Macaques were infused with anti-FasL or the control antibody at 1 week before, the time of, then 1, 2, and 3 weeks after SIV inoculation for a cumulative antibody dose of 20 mg/kg over a period of 5 weeks.

### 4.4. Cytolysis and antibody assays

To measure MHC-unrestricted cytolytic activity, human or monkey effector cells were incubated with chromium-51 (51Cr)-labeled human B-LCL as described [23]. To measure SIV-specific cell-mediated immunity, effectors were macaque PBMC and targets were syngeneic B-LCL (different lines for each animal) that were either uninfected or infected with VVgag as described [26], then labeled with ^51^Cr. Effector PBMC were isolated from the whole blood of uninfected or SIV-infected rhesus monkeys by Ficoll-Hypaque density gradient centrifugation, stimulated in vitro with 5 μg/ml concanavalin A (Sigma, St. Louis, MO) for 3 days followed by culture in 20 U/ml recombinant human interleukin-2 for an additional 4 days. Standard 4-hour chromium-release assays were performed in triplicate in 96-well U-bottom microtiter plates (Costar, Cambridge, MA) as described previously [23]. To determine MHC-unrestricted lysis, assays were done in the presence of 1 mM EGTA to exclude xeno-lysis. Percent specific lysis was determined with the following formula: 100 X (experimental release - spontaneous release)/(maximum release- spontaneous release). Maximum release was determined by the lysis of targets in 1% Triton X-100. Spontaneous release was determined by the lysis of targets in medium without effectors, which was consistently less than 20% of specific lysis.

Virus-binding titers were measured by ELISA using plates coated with p27 Gag antigen as described [27]. Sera from weeks 12 (shaded bars) or 24 (solid bars) were diluted with normal saline, up to a maximum dilution of 1:100,000. Antibodies bound to p27 Gag protein were detected with secondary antibodies linked to horse radish peroxidase.

### 4.5. Flow cytometry for subset identification and for apoptosi*s*

CD4+, CD8+, and CD20+ lymphocyte subsets in PBMC were identified by flow cytometry. $5\times 10^{5}$ PBMC were fixed with 1% paraformaldehyde and stained with 5 ul FITC or PE-conjugated monoclonal antibody against CD4 or CD8 (Antigenix America, Franklin Square, NY), or 10 ul FITC or PE-conjugated anti-CD20 (Becton-Dickinson, Mountain View, CA). Relevant isotype controls were included. Samples were analyzed on a FACScan flow cytometer (Becton-Dickinson), and data were processed using Becton-Dickinson Cell Quest software.

Fixed PBMC that had been already stained with PE-CD20, PE-CD4, or PE-CD8 were also stained for surface FasL using purified antibody against FasL (NOK2) [21] followed by FITC-conjugated goat-anti-mouse IgG. Labeled isotype control (IgG2a) was included. PBMC were labeled for surface markers and then incubated for 20 minutes at 4°C in PBS that contained 20 ug/mL of 7-AAD (Sigma, St. Louis MO). 7-AAD staining was chosen because it is an early indication of cell death that does not interfere with other lymphocyte markers and allows exclusion of cell debris [56]. Samples were washed in PBS + 2% FBS containing 20 ug/mL of nonfluorescent actinomycin D (AD, Sigma) and fixed in the same buffer containing 1% paraformaldehyde. Samples were analyzed 15 minutes later (10,000 events per sample) using the FL-3 channel to detect 7-AAD staining.

### 4.6. Virus burden assays

Virus RNA loads were determined by SIVmac RNA b-DNA assays conducted by Bayer Diagnostics (Emeryville, CA) and reported as copies of SIVmac RNA per mL of EDTA plasma.

## Figures and Tables

**Table 1:** Anti-FasL injection in vivo transiently decreases PBMC cytotoxicity. The values for specific lysis in standard conditions are shown for each specimen at time points between 2 weeks before infection (−2) to 20 weeks after infection. All the PBMC samples shown had mean fluorescent intensity (MFI) levels for FasL ranging from 95–180 as compared to <90 MFI for macaques treated with anti-FasL and sampled between weeks 2 and 10.

Week	−2	2	10	20
RNOK203	24.8	2.1	5.7	22.3
Control IgG	25.2	24.3	22.0	20.2
Untreated	23.8	22.0	21.3	20.9

**Table 2:** Absolute lymphocyte cell counts. Absolute cell counts are given as cells per microliter of blood. Each number is the average of the absolute counts for 4 animals. The counts in parentheses reflect the range of counts seen in 4 animals at that time point. Time is given in weeks from the start of antibody treatment. Week 0 is the lymphocyte count on the first day of treatment, taken before the inoculation with antibody.

Time group	Anti-FasL-treated group			IgG-treated (control)		
	CD4 (range)	CD8 (range)	CD20 (range)	CD4 (range)	CD8 (range)	CD20 (range)
Wk 0	3185 (4228–2512)	3023 (4409–2612)	1233 (2100–640)	2519 (3002–1079)	2246 (3123–1554)	779 (1021–292)
Wk 8	2630 (3965–1872)	2953 (3794–1721)	1206 (2128–536)	773 (1167–641)	2035 (2681–1567)	331 (579–148)
Wk 20	1450 (2441–1066)	2432 (3244–1063)	998(1247–608)	734(1585–620)	1336(2230–945)	227(349–120)

## References

[1] Ameisen JC, Capron A (1991). Cell dysfunction and depletion in AIDS: the programmed cell death hypothesis. *Immunology Today*.

[2] Groux H, Torpier G, Monte D, Mouton Y, Capron A, Ameisen JC (1992). Activation-induced death by apoptosis in ${\text{CD4}}^{\text{+}}$ T cells from human immunodeficiency virus-infected asymptomatic individuals. *Journal of Experimental Medicine*.

[3] Kaplan D, Sieg S (1998). Role of the Fas/Fas ligand apoptotic pathway in human immunodeficiency virus type 1 disease. *Journal of Virology*.

[4] Estaquier J, Tanaka M, Suda T, Nagata S, Golstein P, Ameisen JC (1996). Fas-mediated apoptosis of ${\text{CD4}}^{\text{+}}$
and ${\text{CD8}}^{\text{+}}$
T cells from human immunodeficiency virus-infected persons: differential in vitro preventive effect of cytokines and protease antagonists. *Blood*.

[5] Lenardo MJ (1991). Interleukin-2 programs mouse α
β
T lymphocytes for apoptosis. *Nature*.

[6] Zhou S, Ou R, Huang L, Price GE, Moskophidis D (2004). Differential tissue-specific regulation of antiviral ${\text{CD8}}^{\text{+}}$
T-Cell immune responses during chronic viral infection. *Journal of Virology*.

[7] Zajac AJ, Blattman JN, Murali-Krishna K (1998). Viral immune evasion due to persistence of activated T cells without effector function. *Journal of Experimental Medicine*.

[8] Westendorp MO, Frank R, Ochsenbauer C (1995). Sensitization of T cells to CD95-mediated apoptosis by HIV-1 Tat and gp120. *Nature*.

[9] Uchiyama J, Kishi S, Yagita H, Matsuzaki S, Koga Y (1997). Fas ligand-mediated depletion of CD4 and CD8 lymphocytes by monomeric HIV-1-gp120. *Archives of Virology*.

[10] Yang Y, Dong B, Mittelstadt PR, Xiao H, Ashwell JD (2002). HIV Tat binds Egr proteins and enhances Egr-dependent transactivation of the Fas ligand promoter. *Journal of Biological Chemistry*.

[11] Lichtner M, Maranon C, Vidalain P-O (2004). HIV Type 1-infected dendritic cells induce apoptotic death in infected and uninfected primary ${\text{CD4}}^{\text{+}}$ T lymphocytes. *AIDS Research and Human Retroviruses*.

[12] Jeremias I, Herr I, Boehler T, Debatin K-M (1998). TRAIL/Apo-2-ligand-induced apoptosis in human T cells. *European Journal of Immunology*.

[13] Yang Y, Tikhonov I, Ruckwardt TJ (2003). Monocytes treated with human immunodeficiency virus Tat kill uninfected ${\text{CD4}}^{\text{+}}$ cells by a tumor necrosis factor-related apoptosis-induced ligand-mediated mechanism. *Journal of Virology*.

[14] Esser MT, Bess JW, Suryanarayana K (2001). Partial activation and induction of apoptosis in ${\text{CD4}}^{\text{+}}$ and ${\text{CD8}}^{\text{+}}$ T lymphocytes by conformationally authentic noninfectious human immunodeficiency virus type 1. *Journal of Virology*.

[15] Aquaro S, Panti S, Caroleo MC (2000). Primary macrophages infected by human immunodeficiency virus trigger CD95-mediated apoptosis of uninfected astrocytes. *Journal of Leukocyte Biology*.

[16] Algeciras-Schimnich A, Vlahakis SR, Villasis-Keever A (2002). CCR5 mediates Fas- and caspase-8 dependent apoptosis of both uninfected and HIV infected primary human CD4 T cells. *AIDS*.

[17] Bäumler CB, Böhler T, Herr I, Benner A, Krammer PH, Debatin K-M (1996). Activation of the CD95 (APO-1/Fas) system in T cells from human immunodeficiency virus type-1-infected children. *Blood*.

[18] Badley AD, McElhinny JA, Leibson PJ, Lynch DH, Alderson MR, Paya CV (1996). Upregulation of fas ligand expression by human immunodeficiency virus in human macrophages mediates apoptosis of uninfected T lymphocytes. *Journal of Virology*.

[19] Mitra D (1996). HIV-1 upregulates Fas ligand expression in ${\text{CD4}}^{\text{+}}$ T cells in vitro and in vivo: association with Fas-mediated apoptosis and modulation by aurintricarboxylic acid. *Immunology*.

[20] Sloand EM, Young NS, Kumar P, Weichold FF, Sato T, Maciejewski JP (1997). Role of Fas ligand and receptor in the mechanism of T-cell depletion in acquired immunodeficiency syndrome: effect on ${\text{CD4}}^{\text{+}}$ lymphocyte depletion and human immunodeficiency virus replication. *Blood*.

[21] Oyaizu N, Adachi Y, Hashimoto F (1997). Monocytes express Fas ligand upon CD4 cross-linking and induce ${\text{CD4}}^{\text{+}}$ T cells apoptosis: a possible mechanism of bystander cell death in HIV infection. *Journal of Immunology*.

[22] Monceaux V, Estaquier J, Février M (2003). Extensive apoptosis in lymphoid organs during primary SIV infection predicts rapid progression towards AIDS. *AIDS*.

[23] Yin C, Wu MS, Pauza CD, Salvato MS (1999). High major histocompatibility complex-unrestricted lysis of simian immunodeficiency virus envelope-expressing cells predisposes macaques to rapid AIDS progression. *Journal of Virology*.

[24] Nisihara T, Ushio Y, Higuchi H (2001). Humanization and epitope mapping of neutralizing anti-human Fas ligand monoclonal antibodies: structural insights into Fas/Fas ligand interaction. *Journal of Immunology*.

[25] Regier DA, Desrosiers RC (1990). The complete nucleotide sequence of a pathogenic molecular clone of simian immunodeficiency virus. *AIDS Research and Human Retroviruses*.

[26] Trivedi P, Horejsh D, Hinds SB (1996). Intrarectal transmission of simian immunodeficiency virus in rhesus macaques: selective amplification and host responses to transient or persistent viremia. *Journal of Virology*.

[27] Steger KK, Dykhuizen M, Mitchen JL (1998). ${\text{CD4}}^{\text{+}}$-T-cell and ${\text{CD20}}^{\text{+}}$-B-cell changes predict rapid disease progression after simian-human immunodeficiency virus infection in macaques?. *Journal of Virology*.

[28] Dykhuizen M, Mitchen JL, Montefiori DC (1998). Determinants of disease in the simian immunodeficiency virus-infected rhesus macaque: characterizing animals with low antibody responses and rapid progression. *Journal of General Virology*.

[29] Mattapallil JJ, Douek DC, Hill B, Nishimura Y, Martin M, Roederer M (2005). Massive infection and loss of memory ${\text{CD4}}^{\text{+}}$ T cells in multiple tissues during acute SIV infection. *Nature*.

[30] Li Q, Dua L, Estes JD (2005). Peak SIV replication in resting memory ${\text{CD4}}^{\text{+}}$ T cells depletes gut lamina propria ${\text{CD4}}^{\text{+}}$ T cells. *Nature*.

[31] Finkel TH, Tudor-Williams G, Banda NK (1995). Apoptosis occurs predominantly in bystander cells and not in productively infected cells of HIV- and SIV-infected lymph nodes. *Nature Medicine*.

[32] Geleziunas R, Xu W, Takeda K, Ichijo H, Greene WC (2001). HIV-1 Nef inhibits ASK1-dependent death signalling providing a potential mechanism for protecting the infected host cell. *Nature*.

[33] Ndolo T, Dhillon NK, Nguyen H, Guadalupe M, Mudryj M, Dandekar S (2002). Simian immunodeficiency virus Nef protein delays the progression of ${\text{CD4}}^{\text{+}}$ T cells through G1/S phase of the cell cycle. *Journal of Virology*.

[34] Xu X-N, Screaton GR, Gotch FM (1997). Evasion of cytotoxic T lymphocyte (CTL) responses by nef-dependent induction of Fas ligand (CD95L) expression on simian immunodeficiency virus- infected cells. *Journal of Experimental Medicine*.

[35] Villinger F, Folks TM, Lauro S (1996). Immunological and virological studies of natural SIV infection of disease-resistant nonhuman primates. *Immunology Letters*.

[36] Fultz PN, Stricker RB, McClure HM, Anderson DC, Switz er WM, Horaist C (1990). Humoral response to SIV/SMM infection in macaque and mangabey monkeys. *Journal of Acquired Immune Deficiency Syndromes*.

[37] Hirsch VM (2004). What can natural infection of African monkeys with simian immunodeficiency virus tell us about the pathogenesis of AIDS?. *AIDS Reviews*.

[38] Hurtrel B, Petit F, Arnoult D, Muller-Trutwin M, Silvestri G, Estaquier J (2005). Apoptosis in SIV infection. *Cell Death and Differentiation*.

[39] Enders PJ, Yin C, Martini F (2003). HIV-mediated γσ T cell depletion is specific for $\text{V}\gamma 2^{+}$
cells expressing the Jγ1.2
segment. *AIDS Research and Human Retroviruses*.

[40] Sandberg JK, Fast NM, Palacios EH (2002). Selective loss of innate ${\text{CD4}}^{\text{+}}$ Vα24 natural killer T cells in human immunodeficiency virus infection. *Journal of Virology*.

[41] Brunner T, Mogil RJ, LaFace D (1995). Cell autonomous Fas (CD95)/Fas-ligand interaction mediates activation-induced apoptosis in T-cell hybridomas. *Nature*.

[42] Dhein J, Walczak H, Baumler C, Debatin K-M, Krammer PH (1995). Autocrine T-cell suicide mediated by APO-1/(Fas/CD95). *Nature*.

[43] Ju S-T, Panka DL, Cui H (1995). Fas(CD95)/FasL interactions required for programmed cell death after T cell activation. *Nature*.

[44] Alderson MR, Tough TW, Davis-Smith T (1995). Fas ligand mediates activation-induced cell death in human T lymphocytes. *Journal of Experimental Medicine*.

[45] Lelièvre JD, Mammano F, Arnoult D (2004). A novel mechanism for HIV1-mediated bystander ${\text{CD4}}^{\text{+}}$ T-cell death: neighboring dying cells drive the capacity of HIV1 to kill noncycling primary ${\text{CD4}}^{\text{+}}$ T cells. *Cell Death and Differentiation*.

[46] Stohl W, Xu D, Starling GC, Casali P, Kiener PA (2000). Promotion of activated human B cell apoptosis and inhibition of Ig production by soluble CD95 ligand: CD95-based downregulation of Ig production need not culminate in activated B cell death. *Cellular Immunology*.

[47] Rothstein TL, Wang JKM, Panka DJ (1995). Protection against Fas-dependent Th1-mediated apoptosis by antigen receptor 
engagement in B cells. *Nature*.

[48] Tsai C-C, Emau P, Sun JC (2000). Post-exposure chemoprophlaxis (PECP) against SIV infection of macaques as a model for protection from HIV infection. *Journal of Medical Primatology*.

[49] Smith MS, Foresman L, Lopez GJ (2000). Lasting effects of transient postinoculation tenofovir [9-R-(2-phosphonomethoxypropyl)adenine] treatment on SHIV(KU2) infection of rhesus macaques. *Virology*.

[50] Spring M, Stahl-Hennig C, Stolte N (2001). Enhanced cellular immune response and reduced ${\text{CD8}}^{\text{+}}$ lymphocyte apoptosis in acutely SIV-infected rhesus macaques after short-term antiretroviral treatment. *Virology*.

[51] Rosenberg ES, Altfeld M, Poon SH (2000). Immune control of HIV-1 after early treatment of acute infection. *Nature*.

[52] Kaufmann DE, Lichterfeld M, Altfeld M (2004). Limited durability of viral control following treated acute HIV infection. *PLoS Medicine*.

[53] Pilcher CD, Fiscus SA, Nguyen TQ (2005). Detection of acute infections during HIV testing in North Carolina. *New England Journal of Medicine*.

[54] Salvato MS, Emau P, Malkovsky M, Schultz KT, Johnson E, Pauza CD (1994). Cellular immune responses in rhesus macaques infected rectally with low dose simian immunodeficiency virus. *Journal of Medical Primatology*.

[55] Kayagaki N, Kawasaki A, Ebata T (1995). Metalloproteinase-mediated release of human Fas ligand. *Journal of Experimental Medicine*.

[56] Lecoeur H, Ledru E, Prévost M-C, Gougeon M-L (1997). Strategies for phenotyping apoptotic peripheral human lymphocytes comparing ISNT, annexin-V and 7-AAD cytofluorometric staining methods. *Journal of Immunological Methods*.

